# Psychological Care Needs and Mental Health Service Use Among Adults with Diabetes: Evidence from the Diabetes, Distress, and Disparities (3D) Study

**DOI:** 10.3390/healthcare13121427

**Published:** 2025-06-14

**Authors:** Briana Mezuk, Kara M. Mannor, Rebecca Hebert, Lauren Kouassi, Bella Flores, Emma Spring, Alejandro Rodríguez-Putnam

**Affiliations:** 1 Department of Epidemiology, University of Michigan School of Public Health, Ann Arbor, MI 48109, USA; kmannor@umich.edu (K.M.M.); beccaheb@umich.edu (R.H.); lkouassi@umich.edu (L.K.); bellaflo@umich.edu (B.F.); sprinemm@umich.edu (E.S.); arputnam@umich.edu (A.R.-P.); 2 Research Center for Group Dynamics, Institute for Social Research, Ann Arbor, MI 48106, USA

**Keywords:** diabetes, depression, anxiety, diabetes-related distress, mental health services

## Abstract

Background/Objectives: Mental disorders and diabetes-related distress (DRD) are under-addressed aspects of person-centered diabetes care. This study examines the burden of depression, anxiety, and DRD among adults with type 1 (T1D), latent autoimmune diabetes in adults (LADA), type 2 (T2D), and gestational diabetes (GD), and explores their experiences and barriers in receiving mental health services. Methods: This study uses quantitative data from the 2023/24 Diabetes, Distress, and Disparities (3D) Study, which is based at a large US medical center. The 3D Study consists of 573 adults with diabetes (51.3% with T1D or LADA, 43.5% with T2D, and 4.4% with current/past GD). Mental health assessments included the Patient Health Questionnaire-9 (depression), Generalized Anxiety Disorder-7 (anxiety), and Problem Areas in Diabetes-11 (DRD). Logistic regression was used to examine the prevalence of mental health concerns and behavioral service use. Results: Overall, 14.5% had clinically significant depression, 8.0% had anxiety, and 23.6% had elevated DRD. Symptoms of depression, anxiety, and DRD had a positive, non-linear relationship with poor glycemic control. Approximately 30% of those with clinically significant emotional health concerns did not receive any behavioral health services in the past 12 months. Black adults were less likely to receive behavioral health treatment than non-Hispanic Whites (Odds Ratio = 0.24, 95% CI: 0.07–0.77). Common reasons for not receiving behavioral health services included not knowing where to go, cost, and lack of accessible providers. Conclusions: Gaps in addressing the emotional health needs of people with diabetes persist. Healthcare systems need to integrate addressing psychosocial factors as part of person-centered diabetes care.

## 1. Introduction

The psychosocial aspects of diabetes (e.g., mental disorders, emotional distress) are an important, yet under-addressed, feature of living with this condition [1]. It is established that people with diabetes (PWD) have approximately twice the prevalence of anxiety and depression relative to people who do not have diabetes (~20% vs. 10%) [2,3]. The relationship between diabetes and poor mental health is bi-directional, likely reflecting both common biological (i.e., hypercortisolism, inflammation) and behavioral (i.e., disrupted sleep, poor diet, alcohol and tobacco use) mediating pathways [4,5]. This bi-directional relationship varies by diabetes type. Type 2 diabetes (T2D) typically does not onset until the early 60s, and, as a result, depression and anxiety (which have highest incidence in adolescence and early adulthood) are more often predictors, rather than consequences, of a new T2D diagnosis [4,6]. In contrast, depression and anxiety are often concurrent with, or sequelae of, a diagnosis of type 1 (T1D) [7,8]. While less well studied, perinatal depression and anxiety can also accompany a diagnosis of gestational diabetes (GD) [9,10]. Finally, regardless of diabetes type, depression and anxiety are both predictors and correlates of poor glycemic control and diabetes-related complications [11].

In addition, between 30 and 50% of PWD experience diabetes-related distress (DRD), which reflects the emotional burden of the daily self-management required of this condition [12,13]. While DRD often co-occurs with mental disorders, leaders in the field argue that it is a distinct psychosocial construct [14,15]. The distinction between DRD and depression and anxiety is warranted, in part, due to the different approaches typically used to manage these emotional states [16]. While clinical trials have demonstrated that psychotherapy is as effective as pharmacotherapy for treating many depression and anxiety disorders, in the US and in many other developed economies, these conditions are typically solely treated with medications [17]. In contrast, the interventions for DRD are largely non-pharmacologic and often involve cognitive behavioral therapy and even mindfulness practices to help PWD manage their concerns and develop self-efficacy around self-care behaviors [18,19,20]. Moreover, given how many PWD experience DRD, it has been argued that addressing negative emotions around living with diabetes should be understood as a “regular part of diabetes care” rather than a “comorbidity” or “complication” of diabetes, which is how depression and anxiety are often understood in this context [21].

Regardless of whether mental health concerns occur before, after, or alongside a diagnosis of diabetes, these psychosocial factors have important implications for “person-centered” diabetes care, i.e., care that centers a PWD’s goals, needs, and values regarding their health [22]. The importance and relevance of psychosocial factors to person-centered diabetes care were first enumerated by the Diabetes Attitudes, Wishes and Needs (DAWN) Studies. These multi-national studies surveyed thousands of adults with T1D and T2D, as well as healthcare providers, and provided comprehensive assessments of the impact of diabetes on people’s lives [23,24]. DAWN illustrated that PWD report that their disease negatively impacts multiple domains of their lives, including their overall physical health (62%), relationships with family/friends (20%), and emotional well-being (46%) [13]. Despite this broad impact, DAWN found that less than one-third of PWD report that their healthcare provider asked about their emotional health or how diabetes affects their lives in the past 12 months; in contrast, nearly 75% say their healthcare team measured their blood sugar during this same period. Follow-up studies of DAWN showed that less than 20% of PWD with clinically significant depression and/or DRD received any form of care for these symptoms [25]. Collectively, DAWN illustrated the substantial gap between the psychosocial aspects of diabetes care that PWD report needing and the actions and structures of the healthcare systems they must navigate [26].

Since the completion of the DAWN studies in 2012, the American Diabetes Association (ADA) has made two major efforts to address the concerns they raised. The first was a position statement in 2016 which argued that addressing psychological well-being was essential to the delivery of “patient”-centered care, calling for collaborative, integrated psychosocial care to be provided (scored as an “A” recommendation), and that providers should screen for DRD, depression, anxiety, and related behaviors multiple times over the course of the condition (scored as a “B” recommendation) [1]. In 2020, the ADA released its Behavioral Health Toolkit, which provided both decision guidance and standardized assessment tools for healthcare providers to implement these recommendations [27]. In sum, over the past decade there has been increasing attention to the psychosocial care needs of PWD by both researchers and professional organizations. Therefore, it is reasonable to assess whether the care gaps identified by DAWN have decreased.

In addition, the advent of diabetes management technology (e.g., continuous glucose monitors) and related mobile technology devices (e.g., monitors for actigraphy, sleep, mood) mean that PWD potentially have access to a wide array of digital data on their overall health, and their diabetes management specifically, which was not true when the DAWN studies were fielded. However, such devices remain cost-prohibitive for many people, and, even among those who have access, it is an open question as to how this technology ecosystem can support (or even impair) the emotional aspects of diabetes management [28,29]. Moreover, mental/behavioral health care remains challenged by understaffing, long wait times, and a lack of culturally appropriate providers. Collectively, this suggests that under-detection and under-treatment of mental health conditions and DRD among PWD may persist despite efforts by the ADA for reform [30].

Building on this large body of research on the psychosocial aspects of diabetes, the goals of this paper are threefold: First, to describe the burden of clinically significant depression, anxiety, and DRD among a diverse sample of adults with T1D, latent autoimmune diabetes in adulthood (LADA), T2D, and GD. Second, to examine the experiences of mental health service utilization among these individuals seen at a large US-based medical center. Finally, to characterize the barriers experienced by adults with diabetes when they seek mental health services. By mapping the mental health care needs among PWD, this project can help identify actionable gaps that health systems can address in order to provide more person-centered diabetes care.

## 2. Materials and Methods

Sample. Participants come from the Diabetes, Distress, and Disparities (3D) Study of Self-Management and Mental Health, which was fielded over an 18-month period in 2023–2024. The primary objective of the 3D Study is to quantify and characterize the psychosocial care needs of adults with T1D, LADA, T2D, and current or past GD. The overarching goal of the project is to partner with various stakeholders to implement evidence-based strategies to address identified gaps in person-centered diabetes care in the Michigan Medicine healthcare system and beyond.

The 3D Study employed a two-part sequential mixed-methods design: Part 1 consisted of a 30–40-min quantitative web survey (in Qualtrics) that assessed a range of topics relevant to self-management including diabetes-related distress, depression, anxiety, diabetes-related stigma, technology use, social support, and social determinants of health. Part 1 consists of 573 PWD (5% overall response rate). Details of the data collection and fieldwork procedures are described elsewhere [31]. Briefly, persons aged 18+ with a current diagnosis or history of diabetes seen at Michigan Medicine in the prior two years were identified by ICD-9/10 codes: 249, 250, 775.1, E08, E09, E10, E11, E13, O24, and P70.2. People with T1D and GD were over-sampled to increase their representation, given the relatively low prevalence of these forms of diabetes relative to T2D. Individuals were invited via email to participate in the survey by an intermediate Michigan Medicine Electronic Health Record (EHR) broker to protect confidentiality. Participants who completed the Part 1 survey and indicated their willingness to be contacted about future research were eligible for Part 2 of the study, which consisted of a 60-min one-on-one semi-structured interview to probe more complex topics such as patient–provider relationships, priorities in care, and self-efficacy (*n* = 40 also completed Part 2). This analysis only uses data from Part 1 of the 3D Study. This quantitative survey was designed to provide valuable data on the current burden of the three most common mental health concerns for people with T1D/LADA, T2D, and GD, which is essential to informing implementation of healthcare quality improvement efforts.

The 3D Study was approved by the IRB at the University of Michigan (HUM00223735), and all participants provided written informed consent. The 3D Study materials and data collection protocols are publicly available at the Open Science Framework (https://osf.io/yfz6b/, accessed on 24 March 2025).

Depression. Current depressive symptoms were assessed using the Patient Health Questionnaire (PHQ-9), a brief instrument for assessing clinically relevant depression in primary care settings [32]. The PHQ-9 assesses the frequency of nine symptoms drawn from the Diagnostic and Statistical Manual of Mental Disorders (DSM) criteria for Major Depression (i.e., dysphoria, anhedonia, sleep disturbances, appetite disturbances, cognitive challenges, fatigue, psychomotor changes, feeling of worthlessness, and suicidal ideation) each assessed on a 4-point scale ranging from Not at all to Nearly every day, referring to the past 2 weeks. A final item, which asks about how much difficulty the person has experienced due to these symptoms, is not used in the scoring. Only participants with complete data on all nine symptoms were included in the analysis (*n* = 77 due to missing data on one or more symptoms). Given that PWD have more emotional distress symptoms than a general “healthy” population, we chose to apply the more stringent thresholds (e.g., moderately severe or greater) on all psychosocial scales to indicate clinically significant symptoms [2,3]. Therefore, following the instructions of the scale creators, scores were summed (range: 0 to 27) and dichotomized, with values ≥ 15 indicating moderately severe or more depression [32].

Anxiety. Current anxiety symptoms were assessed using the Generalized Anxiety Disorder-7 (GAD-7), a brief screening instrument for assessing clinically relevant anxiety in primary care settings [33]. It consists of seven items drawn from DSM criteria for anxiety disorders (i.e., feeling nervous, uncontrollable worry, general nervousness, being unable to relax, feeling restless, being irritable, and feeling fearful about the future), each assessed on a 4-point scale ranging from Not at all to Nearly every day, referring to the past 2 weeks. A final item, which asks about how much difficulty the person has experienced due to these symptoms, is not used in the scoring. Only participants with complete data on all seven symptoms were included in the analysis (*n* = 58 due to missing data on one or more symptoms). Following the instructions of the scale creators, scores were summed (range: 0 to 21) and dichotomized, applying their threshold of values ≥ 15 to indicate severe anxiety [33].

Diabetes-related distress. Current DRD was assessed using the Problem Areas in Diabetes (PAID-11), a brief instrument for assessing the emotional aspects of living with diabetes [34]. It asks “how much of a current problem” various experiences, feelings, and beliefs related to diabetes are for the person (i.e., 11 items including feeling scared (depressed) about diabetes, uncertainty about emotions, worrying about low blood sugar, concerns about food, worrying about complications, feeling anxiety and/or guilt about self-management, feeling overwhelmed, mental energy needed to manage diabetes, coping with complications, and feeling “burned out”). Each item is assessed on a five-point scale ranging from Not a problem to Serious problem. Only participants with complete data on all 11 items were included in the analysis (*n* = 52 due to missing data on one or more symptoms). Applying the instructors of the scale creators, scores were summed (range: 0 to 44) and dichotomized, applying their threshold of values > 18 indicating clinically relevant DRD [34].

Mental health services use. Use of mental, behavioral, and/or substance abuse services in the past 12 months was assessed by a series of questions drawn from the National Survey on Drug Use and Health [35]. Participants were asked if they had “received any professional counseling, medication, or treatment” for (a) “emotions, mental health, or behavior” and for (b) “alcohol or drug use.” An additional question asked about “stay[ing] overnight or longer in a hospital or other facility to receive treatment or counseling” for “emotions, nerves, or mental health or for drug or alcohol use.” These items were combined into a single dichotomous variable for analysis, indicating any vs. no mental health services use in the past 12 months.

Barriers to mental health services. To assess barriers to receipt of mental health care, participants were asked if there was “any time [in the past 12 months] when you needed mental health treatment or counseling for yourself but didn’t get it?”, and those who responded affirmatively were asked why they were unable to obtain the treatment they needed. Response options were not mutually exclusive and included cost, concern that treatment would cause others to have a negative opinion of them, concern that treatment would have a negative impact on their job, lack of or inadequate insurance coverage, concerns about confidentiality, concerns that they would be forced to undergo inpatient or medication treatment, unable to find a provider, or some other reason. *Diabetes characteristics*. Diabetes type and hemoglobin A1c (HbA1c) were assessed by self-reporting. Individuals who reported a history of multiple types of diabetes were re-coded as having a single “primary” type based on which was more persistent (e.g., individuals who reported both T2D and gestational diabetes were coded as having T2D). Individuals who reported having both T1D and T2D were coded as having LADA, as this diagnosis is often made after a mis-diagnosis of T2D. HbA1c is an indicator of glycemic control over the past 30–90 days, with values < 7% indicating adequate control. Participants were asked to report their most recent HbA1c (%) value; individuals who reported a range (e.g., “usually between 5.6 to 5.9%”), rather than a single value, were assigned the highest value they provided.

Covariates. All covariates were assessed by self-reporting. Demographic characteristics included age (in years), gender (man, woman, gender non-conforming), race/ethnicity (Native American/Alaskan Native, Asian, Black/African American, Latino, White, more than one race, and prefer not to say, which was re-coded for analysis as White, Black/African American, and all others), highest level of education obtained (recoded for analysis as high school or less, associate’s degree/certificate program, bachelor’s degree, and advanced degree), and household income (re-coded for analysis into four income brackets: <USD 50,000, USD 50,000 to <USD 100,000, USD 100,000 to <USD 150,000 and USD 150,000 or more).

Analysis. While this is primarily a descriptive analysis, we have three overarching hypotheses: (1) Depression, anxiety, and DRD (and their co-occurrence) will be more prevalent among PWD compared to similar-age general population samples. (2) Even though participants were drawn from a major academic medical center, the majority of those with clinically significant depression, anxiety, and DRD will not have received any mental health services. (3) Reflecting disparities in healthcare access, factors such as age, gender, race/ethnicity, and socioeconomic status will be associated with barriers to mental health service use even after accounting for clinical need as indicated by depression, anxiety, and DRD symptoms.

Initially, sample characteristics as a function of psychosocial outcomes (i.e., elevated symptoms of depression, anxiety, and DRD) were quantified using descriptive statistics. Next, we used multivariable logistic regression models to examine the prevalence of the outcomes of clinically significant depression, anxiety, and DRD. We plotted the predicted probabilities from the logistic regression models separately by diabetes status and HbA1c values. To inform the HbA1c plots, we assessed the relationship between predicted probabilities of the mental health outcomes and HbA1c by calculating Pearson correlation coefficients. The results suggested non-linear associations, so we used locally estimated scatterplot smoothing (LOESS) to visualize these relationships. Next, multivariable logistic regression was used to identify predictors of mental health service use among those with any mental health concern (e.g., among those with clinically significant depression, anxiety, and/or DRD). Finally, bar charts were used to illustrate the most common reasons cited for not receiving mental health care. Due to small cells, people with LADA were combined with those with T1D, and for most analyses there were insufficient numbers of those with GD to estimate regression models.

While this is primarily a descriptive analysis, when making comparisons we use a threshold of *p* < 0.05 to evaluate statistically significant differences, and all *p*-values refer to two-tailed tests.

## 3. Results

As shown by Table 1, the 3D Study consists of 573 adults with diabetes, approximately half with T1D or LADA (*n* = 294, 51.3%) or T2D (*n* = 249, 43.5%); 25 had current or past history of GD (4.3%), and *n* = 5 did not know their diabetes type. This sample had a mean age of 53.3 years (SD: 17.3, range: 19–92), and 54% were women. As shown by Table 2, participants missing data on at least one mental health measure (i.e., PHQ, GAD, and/or PAID) were younger, more likely to have a race/ethnicity other than White or African American, and had completed less education than those with complete data on all three measures; however, they did not differ from those with complete data in terms of gender, household income, or diabetes type.

### 3.1. Burden of Clinically Relevant Depression, Anxiety, and DRD

Overall, 14.5% had clinically significant depression, 8.0% had clinically significant anxiety, and 23.6% had elevated DRD. As expected, depression, anxiety, and DRD were highly correlated (r2 ranged from 0.645 to 0.797), and 39% of those with clinically significant depression also had clinically significant anxiety. As shown by Table 1, having comorbid depression/anxiety was associated with higher HbA1c (7.93% vs. 6.96% for those with neither condition), and approximately 70% of people with elevated depression, anxiety, or both received some form of behavior or mental health treatment in the past 12 months as compared to 30% who had neither depression or anxiety.

**Figure 1:**
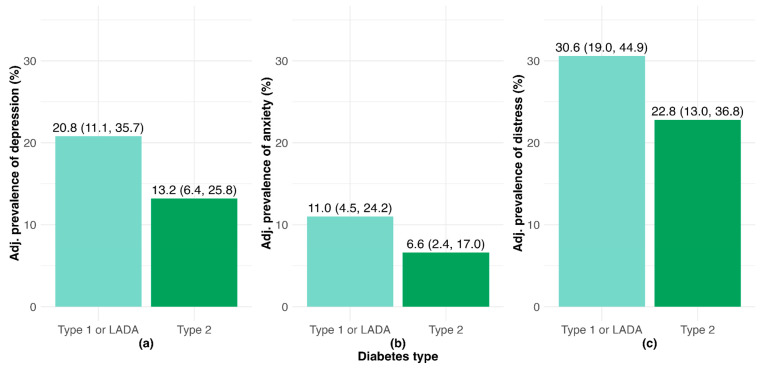
Prevalence values for adults with diabetes adjusted for age, gender, race/ethnicity, income, and education for clinically significant (**a**) depression; (**b**) anxiety; (**c**) diabetes-related distress.

As shown by the panels of Figure 1, adults with T1D or LADA had higher prevalence of clinically significant depression, anxiety, and DRD than those with T2D. Other factors significantly associated with poor mental health included younger age, being a woman, and lower household income; race and educational attainment were not significantly correlated with clinically significant depression, anxiety, or DRD. While not shown in the figure due to small cells, people with current or past GD had relatively low unadjusted prevalence of depression (4.0%), anxiety (4.0%), and DRD (12.0%), indicating that this population may not have the same mental health concerns as other PWD.

**Figure 2:**
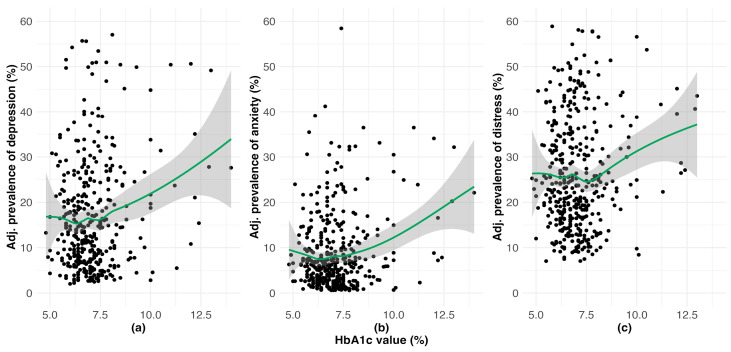
Prevalence values by HbA1c values for adults with diabetes adjusted for age, gender, race/ethnicity, income, and education for clinically significant (**a**) depression; (**b**) anxiety; (**c**) diabetes-related distress.

As shown by the panels of Figure 2 and confirmed by quantitatively estimating the Pearson’s correlation coefficients (r2), there was a non-linear relationship between HbA1c and poor mental health. For HbA1c values < 7.5%, there was no correlation between these measures (depression: r2 = 0.004, 95% Confidence Interval (CI) = –0.11 to 0.12, *p*-value = 0.940; anxiety: r2 = –0.004, 95% CI = –0.12 to 0.11, *p*-value = 0.950; and DRD: r2 = –0.041, 95% CI = –0.15 to 0.07, *p*-value = 0.470). However, for values of HbA1c > 7.5%, higher levels of HbA1c were positively associated with probability of clinically significant depression (r2 = 0.238, 95% CI = 0.04 to 0.42, *p*-value = 0.020), anxiety (r2 = 0.369, 95% CI = 0.18 to 0.53, *p*-value<0.001), and DRD (r2 = 0.342, 95% CI = 0.16 to 0.51, *p*-value < 0.001).

### 3.2. Mental Health Services Use and Barriers to Psychosocial Care Among PWD

Overall, 42.6% had used any form of mental/behavioral health services in the prior 12 months. Unsurprisingly, a greater proportion of people with elevated scores of depression (77.1%), anxiety (76.1%), or DRD (63.0%) received any mental health care in the past year compared to people who had none of these (34.7%). As shown by Table 3, among those with clinically elevated depression, anxiety, and/or DRD (*n* = 153), the only significant predictors of receiving mental health care in the past year were gender, race/ethnicity, and degree of impairment due to symptoms. As expected, people who reported more impairment from their depression/anxiety symptoms were more than twice (Odds Ratio (OR): 2.87, 95% Confidence Interval (CI); 1.30–6.65) as likely to report using mental health services. Women were approximately twice as likely as men to report mental health service use in the past 12 months, which became only marginally statistically significant after accounting for impairment. Black/African American respondents had 76% lower odds of receiving care compared to White respondents, a disparity that persisted even after accounting for symptom impairment. While not shown in the regression table, 66.7% of people with GD who had any clinically significant emotional health concern received any mental health services in the past year.

**Figure 3:**
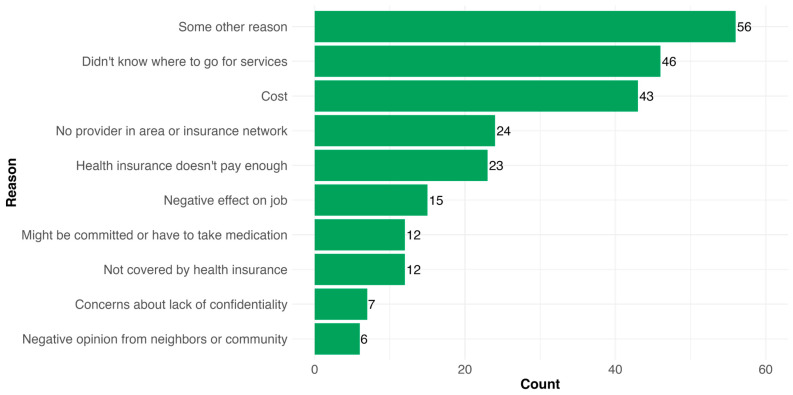
Barriers reported as to why PWD could not access mental health services when needed (responses are not mutually exclusive; *n* = 133).

Finally, Figure 3 shows the most prevalent barriers to mental health service use. While these responses were not mutually exclusive, most people indicated they experienced at least one barrier that was not captured in the list provided. However, among those who listed “some other reason” (*n* = 56), 32.1% also reported not knowing where to go, 8.9% reported a cost barrier, and 10.7% reported issues related to insurance coverage. Among those who indicated a listed reason, the most common responses were related to access and availability (i.e., cost, did not know where to go, lack of in-network providers). Among this group that reported barriers, 48.1% (*n* = 64) reported more than one; the primary factor associated with reporting multiple barriers for receiving mental health services when needed was lower income (54.1% of people with household incomes <USD 50,000 reported > 1 barrier vs. only 2.7% of those with incomes >USD 150,000).

## 4. Discussion

This cross-sectional study of adults with diabetes aimed to describe the “emotional side of diabetes” and use of behavioral health services in this population, in order to understand the extent to which recent efforts by the ADA and related organizations have begun to close gaps in “person-centered” diabetes care [36]. The findings from this study are threefold: First, depression, anxiety, and DRD are highly prevalent in this population and are more common among persons with T1D or LADA compared to those with T2D. Overall, 38.6% of people with T1 or LADA, 23.5% of people with T2D, and 13.6% with GD had at least one mental health concern. For comparison, a 2025 study from the National Health and Nutrition Examination Survey, which also used the PHQ-9 but applied a lower scoring threshold of >9 (vs. our threshold of >14) symptoms, reported the prevalence of depression among US adults was 12.4% [37]. Also, in 2019, the National Health Interview Survey, which also used the GAD-7 and applied the same threshold as the 3D Study, reported that only 2.8% of US adults had clinically significant anxiety [38].

Second, approximately 70% of PWD with clinically significant depression, anxiety or DRD had used some form of behavioral health services in the past year. This is notably higher than previous studies of mental health service use among PWD (i.e., the DAWN studies reported that only 17% of cases of clinically significant depression or DRD among PWD were currently received services [25], and INTERPRET-DD reported that, depending on the site, only between 0% and 30% of people with T2D and comorbid depression had even been clinically identified, to say nothing of treated [39]). While this may indicate a genuine increase in use of mental health services among PWD (consistent with the increased awareness of the need to address psychosocial concerns via the ADA efforts described earlier), given limitations in the sampling (described more below) this may also reflect selection bias. We also found notable disparities in mental health services use. Specifically, Black PWD were significantly less likely to have received any form of behavioral health care, even after accounting for degree of impairment. While other factors also likely contribute (e.g., fragmentation of care), the most commonly cited reasons for not accessing behavioral health services when needed reflected issues of accessibility and/or availability (e.g., not knowing where to go for services, cost, or a lack of providers in the insurance network). Finally, and consistent with prior studies [11], these emotional aspects of diabetes were correlated with diabetes self-management, indicated by glycemic control. Symptoms of depression, anxiety, and DRD were associated with HbA1c in a non-linear manner: while there was no correlation between this indicator and emotional symptoms for A1c values ≤ 7.5%, for A1c values > 7.5% there was a positive association between burden of emotional symptoms and values of A1c.

It is important to recognize that barriers to psychosocial care do not have to be absolute (e.g., zero behavioral health providers in the area) in order to significantly impact the lives of PWD. Standard models of diabetes care tend to distinguish between “disease” and “life”, and this divide creates tensions that have direct implications for the emotional side of this condition [40]. Empirical research has shown that this divide impairs problem-solving even around “disease” topics like medication adherence [41]. Fortunately, decades of empirical health services research have provided compelling evidence that person-centered Collaborative Care Models (CCMs, e.g., models of care in which a team of health professionals with differing expertise, roles, and responsibilities) result in clinically significant improvements in both glycemic control and depressive symptoms for adults with comorbid depression and diabetes [42]. These models of care are also cost-effective [43,44,45]. However, implementation of these models remains low [46], with factors such as financing and staffing needs cited as major barriers [47,48].

Regardless of whether psychosocial support directly improves clinical diabetes indicators [49,50,51], it is established that such interventions are effective for reducing symptoms of anxiety, depression, and DRD and improving quality of life for PWD [49,52,53]. This is an end worthy of pursuit in and of itself for PWD and their families [1]. Given the behavioral health workforce shortage, it is essential that healthcare systems explore creative means of achieving this goal, including employing Community Health Workers (CHWs) or connecting with local organizations (e.g., YMCA, church health ministries, etc.) to implement diabetes peer support programs. Such programs are clinically- and cost-effective means of bridging the gap between “disease” and “life” for PWD in a manner that can be more adaptable to the needs of disadvantaged groups that have a higher burden of diabetes, particularly T2D (e.g., lower income, racial/ethnic minorities, rural areas) [54,55,56,57,58,59,60,61]. It remains to be seen whether technology, which has become such a core part of self-management for PWD (e.g., insulin pump, CGMs, apps, etc.) [62], can also help support the emotional aspects of this condition [63,64]. Future analyses of the 3D Study data can explore this possibility.

The findings should be interpreted in light of the study limitations. First, this is a cross-sectional and primarily descriptive study that cannot test hypotheses of causal relationships. Second, while the PHQ-9, GAD-7, and PAID-11 are widely used screening instruments for depression, anxiety, and DRD, respectively, they are not as sensitive as clinical diagnostic assessments. Third, the 3D Study represents just a single snapshot in time in the lives of PWD, and the limited sample size precluded more sophisticated statistical analyses, particularly of people with GD for whom only basic descriptive analyses were justifiable. However, these quantitative findings set the stage for future work analyzing the one-on-one qualitative interviews (part 2 of the 3D Study) that will explore how PWD experience their condition and navigate mental health services in more depth. Fourth, while web surveys have a significantly lower response rate than other modalities, the low response rate for this study in particular means that we must be cautious when extrapolating the results to the broader population of PWD [65]. Fifth, while we are in the process of linking these surveys to clinical records to obtain clinical data, the measure of HbA1c used in this analysis was based on self-reporting and is thus subject to recall bias. Finally, due to hospital policies protecting patient confidentiality, we are unable to assess the degree to which those who participated in the 3D Study differed from those who were invited but did not respond. This limits our ability to generalize the results to the larger population of PWD seen in this healthcare system or elsewhere; however, we note that the amount of missingness on psychosocial outcomes did not vary by diabetes type, and the prevalence estimates of depression, anxiety, and DRD were in line with prior studies. Given these limitations, these results should be situated within the larger literature (which, we note, they are largely consistent with) rather than providing robust evidence in and of themselves.

This study also has several strengths. Building on the foundational work and insights of the DAWN studies, which highlighted the psychosocial care needs and gaps in person-centered care globally, our study provides a current view of these challenges within a U.S. context. Our data also offer a nuanced look at a range of emotional problems (i.e., anxiety, depression, DRD), including how they are linked to indicators of self-management. By oversampling individuals with T1D or LADA, who comprised a smaller proportion of DAWN2 participants (e.g., only 80 with T1D vs. 420 with T2D per country), we were able to identify important subgroup differences in emotional burden and service use. Finally, the study team consulted with multiple patient advisory boards in the development of the survey instrument to help ensure this study reflects the priorities of the people who live with diabetes [66].

## 5. Conclusions

Despite increased attention to the emotional side of diabetes over the past decade, collectively, our findings emphasize that substantial gaps remain in effectively addressing the emotional aspects of diabetes. Overall, one-third of respondents in the 3D Study were experiencing at least one clinically significant mental health concern at the time of data collection. Meeting the challenge of addressing these psychosocial aspects of diabetes as a “standard” component of diabetes care will likely require substantial revision of current practices [21]. While the CCM and related approaches are a clinically- and cost-effective embodiment of person-centered diabetes care, they are not cost-free [67]. Indeed, no intervention, no matter how minor, that a healthcare system delivers is “free,” and the only ways systems can reduce costs (in an absolute sense) is by reducing demand for services—or by limiting the services they provide—from their patients. The psychosocial aspects of diabetes require a different type of algebra for understanding the costs of (in)action than what is typically employed by providers, systems, and payers. Even if addressing the mental health concerns of PWD (whether through medications or psychotherapy) does not directly impact diabetes clinical outcomes (e.g., HbA1c), this should not discount the benefit to the individual and their family of effectively addressing their emotional needs [68]. Currently, gaps in addressing psychosocial aspects of diabetes care increases the (emotional) costs of living with diabetes [1,69], which has substantial impacts on individuals’ quality of life, ability to work, financial stability, and overall health [70,71]. Healthcare systems should consider that, when they do not provide person-centered diabetes care, they are likely generating costs for PWD, i.e., the very people they seek to serve.

## Figures and Tables

**Table 1:** Characteristics of 3D Study participants by depression, anxiety, and diabetes-related distress status.

	Overall Sample	Depression	Anxiety	Diabetes-Related Distress
N	573	496	515	521
Prevalence of mental health problem	--	83 (14.5%)	46 (8.0%)	135 (23.6%)
Diabetes type				
Type 1 or LADA	294 (51.3%)	48 (57.8%)	25 (54.3%)	88 (65.2%)
Type 2	249 (43.5%)	33 (39.8%)	20 (43.5%)	43 (31.9%)
Gestational (past or current)	25 (4.4%)	1 (1.2%)	1 (2.2%)	3 (2.2%)
Diabetes—Type unknown	5 (0.9%)	1 (1.2%)	0 (0%)	1 (0.7%)
Latest HbA1c value				
Mean (SD)	7.09 (1.38)	7.58 (1.70)	7.81 (1.60)	7.67 (1.64)
Missing	88 (15.4%)	12 (14.5%)	10 (21.7%)	16 (11.9%)
Current age				
Mean (SD)	53.3 (17.3)	45.9 (14.6)	42.7 (14.2)	48.0 (16.4)
Gender				
Man	192 (33.5%)	17 (20.5%)	8 (17.4%)	38 (28.1%)
Woman	307 (53.6%)	60 (72.3%)	34 (73.9%)	92 (68.1%)
Gender non-conforming	4 (0.7%)	2 (2.4%)	2 (4.3%)	1 (0.7%)
Missing	70 (12.2%)	4 (4.8%)	2 (4.3%)	4 (3.0%)
Race/ethnicity				
Native American or Alaskan Native	4 (0.7%)	1 (1.2%)	1 (2.2%)	2 (1.5%)
Asian	15 (2.6%)	0 (0%)	0 (0%)	2 (1.5%)
Black or African American	49 (8.6%)	6 (7.2%)	4 (8.7%)	17 (12.6%)
Latino	9 (1.6%)	1 (1.2%)	1 (2.2%)	1 (0.7%)
White	402 (70.2%)	67 (80.7%)	34 (73.9%)	101 (74.8%)
Prefer not to disclose	83 (14.5%)	5 (6.0%)	4 (8.7%)	9 (6.7%)
More than one race	11 (1.9%)	3 (3.6%)	2 (4.3%)	3 (2.2%)
Highest grade of school				
High school diploma or below	109 (19.0%)	18 (21.7%)	11 (23.9%)	34 (25.2%)
Associate’s degree or certificate program	91 (15.9%)	19 (22.9%)	13 (28.3%)	25 (18.5%)
Bachelor’s degree	188 (32.8%)	24 (28.9%)	10 (21.7%)	35 (25.9%)
Advanced degree	21 (3.7%)	22 (26.5%)	12 (26.1%)	41 (30.4%)
Annual household income				
Less than USD 50,000	173 (30.2%)	42 (50.6%)	25 (54.3%)	56 (41.5%)
USD 50,000–USD 99,999	174 (30.4%)	17 (20.5%)	8 (17.4%)	39 (28.9%)
USD 100,000–USD 149,999	92 (16.1%)	12 (14.5%)	7 (15.2%)	20 (14.8%)
USD 150,000 or more	101 (17.6%)	12 (14.5%)	6 (13.0%)	19 (14.1%)
Missing	33 (5.8%)	0 (0%)	0 (0%)	1 (0.7%)
Behavioral or mental health treatment in past 12 months				
Yes	206 (36.0%)	59 (71.1%)	33 (71.7%)	70 (51.9%)
No	324 (56.5%)	24 (28.9%)	13 (28.3%)	63 (46.7%)
Missing	43 (7.5%)	0 (0%)	0 (0%)	2 (1.5%)
Take prescription medication for mental/emotional condition in the past 12 months				
Yes	195 (34.0%)	59 (71.1%)	32 (69.6%)	71 (52.6%)
No	329 (57.4%)	22 (26.5%)	13 (28.3%)	58 (43.0%)
Missing	49 (8.6%)	2 (2.4%)	1 (2.2%)	6 (4.4%)
Any mental health treatment or prescription medication use for mental health condition in last 12 months				
Yes	296 (51.7%)	64 (77.1%)	35 (76.1%)	85 (63.0%)
No	244 (42.6%)	19 (22.9%)	11 (23.9%)	50 (37.0%)
Missing	33 (5.8%)	0 (0%)	0 (0%)	0 (0%)
PHQ-9 score				
Mean (SD)	7.68 (6.33)	18.7 (2.80)	18.9 (4.43)	13.2 (5.97)
Missing	77 (13.4%)	0 (0%)	9 (19.6%)	9 (6.7%)
PAID-11 score				
Mean (SD)	11.8 (9.97)	23.0 (9.81)	24.8 (10.3)	25.9 (6.16)
Missing	52 (9.1%)	1 (1.2%)	0 (0%)	0 (0%)
GAD-7 score				
Mean (SD)	5.28 (5.49)	13.0 (4.94)	17.8 (2.02)	10.2 (5.81)
Missing	58 (10.1%)	4 (4.8%)	0 (0%)	3 (2.2%)
Difficulty at work, home, or in relationships due to PHQ-9 problems				
Not at all difficult	229 (40.0%)	2 (2.4%)	1 (2.2%)	15 (11.1%)
Somewhat difficult	189 (33.0%)	41 (49.4%)	17 (37.0%)	75 (55.6%)
Very difficult	47 (8.2%)	23 (27.7%)	14 (30.4%)	29 (21.5%)
Extremely difficult	22 (3.8%)	16 (19.3%)	14 (30.4%)	14 (10.4%)
Missing	86 (15.0%)	1 (1.2%)	0 (0%)	2 (1.5%)
Difficulty at work, home, or in relationships due to GAD-7 problems				
Not at all difficult	264 (46.1%)	6 (7.2%)	0 (0%)	17 (12.6%)
Somewhat difficult	201 (35.1%)	41 (49.4%)	20 (43.5%)	83 (61.5%)
Very difficult	41 (7.2%)	22 (26.5%)	12 (26.1%)	23 (17.0%)
Extremely difficult	17 (3.0%)	13 (15.7%)	14 (30.4%)	12 (8.9%)
Missing	50 (8.7%)	1 (1.2%)	0 (0%)	0 (0%)

Depression: PHQ-9 ≥ 15 (severe or moderately severe); *n* = 77 missing PHQ-9. Anxiety: GAD-7 ≥ 15 (severe); *n* = 58 missing GAD-7. Diabetes-related distress: PAID-11 ≥ 18 (severe); *n* = 52 missing PAID-11.

**Table 2:** Comparison of 3D Study participants with complete vs. incomplete mental health outcome data.

	Complete Data on Mental Health Conditions	Missing Data on at Least One Mental Health Condition	Z or χ^2^, *p*-Value
N	480	93	
Current age			
Mean (SD)	54.2 (17.3)	48.8 (16.6)	2.84, *p* = 0.005
Gender			
Woman	285 (59.4%)	22 (23.7%)	0.20, *p* = 0.660
Missing	16 (3.3%)	54 (58.1%)	
Race/ethnicity *			
Black or African American	44 (9.2%)	5 (5.4%)	106.19, *p* < 0.001
White	371 (77.3%)	31 (33.3%)	
All others	65 (15.7%)	57 (61.3%)	
Highest grade of school			
>High school diploma	392 (81.7%)	51 (54.8%)	3.98, *p* = 0.046
Missing	0%	21 (22.6%)	
Annual household income			
> USD 100,000	173 (30.2%)	20 (21.5%)	1.25, *p* = 0.263
Missing	9 (1.9%)	24 (25.8%)	
Diabetes type			
Type 1 or LADA	246 (51.3%)	48 (51.6%)	*p* = 0.904 **
Type 2	207 (43.1%)	42 (45.2%)	
Gestational (past or current)	22 (4.6%)	3 (3.2%)	
Self-reported latest A1c value			
Mean (SD)	7.05 (1.36)	7.35 (1.47)	−2.23, *p* = 0.028
Missing	9 (2.1%)	2 (2.9%)	

* The race/ethnicity question has a ‘prefer not to disclose’ response category (see Table 1). Missing values for race/ethnicity were grouped into that category, which was then grouped into ‘all others’ for this table. ** Fisher’s exact test.

**Table 3:** Predictors of receiving any mental health services in the prior 12 months among adults with diabetes and clinically significant depression, anxiety, or DRD.

Characteristic	OR	95% CI	*p*-Value	OR	95% CI	*p*-Value
Diabetes type						
Type 2 (ref.)	—	—		—	—	
Type 1/Type 1.5	1.24	0.53, 2.90	0.600	1.35	0.56, 3.24	0.500
Current age	1.00	0.97, 1.03	>0.900	1.01	0.98, 1.03	0.700
Annual household income						
Less than USD 50,000 (ref.)	—	—		—	—	
USD 50,000–USD 99,999	0.57	0.23, 1.39	0.200	0.66	0.26, 1.68	0.400
USD 100,000–USD 149,999	0.40	0.14, 1.17	0.100	0.42	0.14, 1.30	0.130
USD 150,000 or more	1.02	0.30, 3.87	>0.900	1.15	0.32, 4.47	0.800
Gender						
Man (ref.)	—	—		—	—	
Woman	2.43	1.11, 5.40	0.027	2.03	0.89, 4.65	0.093
Highest grade of school						
High school or less (ref.)	—	—		—	—	
Associate’s degree or certificate program	0.61	0.20, 1.79	0.400	0.53	0.17, 1.61	0.300
Bachelor’s degree	0.73	0.26, 2.03	0.600	0.71	0.24, 2.03	0.500
Advanced degree	1.09	0.36, 3.26	0.900	1.10	0.36, 3.41	0.900
Race/ethnicity						
White (ref.)	—	—		—	—	
Black or African American	0.24	0.07, 0.77	0.018	0.26	0.07, 0.86	0.028
Another race	1.28	0.37, 5.19	0.700	1.58	0.44, 6.67	0.5
Difficulty at work, home, or in relationships due to PHQ-9 or GAD-7 problems *						
Somewhat or not difficult at all (ref.)	—	—	—	—	—	
Very or somewhat difficult	—	—	—	2.87	1.30, 6.65	0.011
N	153			151		

Abbreviations: CI = Confidence Interval, OR = Odds Ratio. * Highest value reported for PHQ-9 or GAD-7 difficulty questions.

## Data Availability

The 3D Study materials are publicly available at https://osf.io/yfz6b/ (accessed on 24 March 2025).
